# Prednisolone Trial: Study protocol for a randomised controlled trial of prednisolone for women with idiopathic recurrent miscarriage and raised levels of uterine natural killer (uNK) cells in the endometrium

**DOI:** 10.1186/1745-6215-10-102

**Published:** 2009-11-10

**Authors:** Ai-Wei Tang, Zarko Alfirevic, Mark A Turner, Jo Drury, Siobhan Quenby

**Affiliations:** 1 School of Reproductive and Developmental Medicine, University of Liverpool, Liverpool Women's Hospital, Crown Street, Liverpool, L8 7SS, UK

## Abstract

**Background:**

Idiopathic recurrent miscarriage is defined as 3 consecutive pregnancy losses with no contributing features found on investigations. At present there are no treatments of proven efficacy for idiopathic recurrent miscarriage. Uterine natural killer (uNK) cells, the most predominant leucocyte in the endometrium are adjacent to foetal trophoblast cells and thought to be involved in implantation. The exact mechanisms of how uNK cells affect implantation are not clear but are probably through the regulation of angiogenesis. Multiple studies have shown an association between high density of uterine natural killer cells and recurrent miscarriage. We have shown that prednisolone reduces the number of uNK cells in the endometrium. The question remains as to whether reducing the number of uNK cells improves pregnancy outcome.

**Methods:**

We propose a randomised, double-blind, placebo controlled trial of prednisolone with a pilot phase to assess feasibility of recruitment, integrity of trial procedures, and to generate data to base future power calculations. The primary aim is to investigate whether prednisolone therapy during the first trimester of pregnancy is able to improve live birth rates in patients with idiopathic recurrent miscarriage and raised uNK cells in the endometrium. Secondary outcomes include conception rate, karyotype of miscarriage, miscarriages (first and second trimester), stillbirths, pregnancy complications, gestational age at delivery, congenital abnormality and side effects of steroids. The trial has 2 stages: i) screening of non-pregnant women and ii) randomisation of the pregnant cohort. All patients who fit the inclusion criteria (<40 years old, ≥3 consecutive miscarriages with no cause found and no contraindications to prednisolone therapy) will be asked to consent to an endometrial biopsy in the mid-luteal phase to assess their levels of uNK cells. Women with high levels of uNK cells (≥5%), will be randomised to either prednisolone or placebo when a pregnancy is confirmed. Follow-up includes 2 weekly ultrasound scans in the first trimester, an anomaly scan at 20 weeks gestation, growth scans at 28 and 34 weeks gestation and a postnatal follow-up at 6 weeks.

**Trial Registration:**

Current Controlled Trials ISRCTN28090716

## Background

Recurrent miscarriage (RM) is defined as the loss of 3 or more consecutive pregnancies and is a stressful condition for both patients and clinicians alike. It affects about 1% of all fertile couples trying to conceive [1,2]. Despite a wide range of investigations, no apparent cause is found in more that 50% of cases and they are categorized as idiopathic recurrent miscarriage [2,3]. Apart from supportive care in a dedicated early pregnancy unit with regular reassurance scans and psychological support, empirical treatment in this group of women is not recommended [1,4]. Immunomodulation therapies such as steroids, intravenous immunoglobulin (IVIG), 3rd party donor cell immunization, paternal cell immunization and trophoblast membrane infusion have been proposed with conflicting evidence as to their efficacies. A meta-analysis of trials evaluating IVIG, 3rd party donor cell immunization, paternal cell immunization and trophoblast membrane infusion found no evidence of a beneficial effect over placebo in preventing further miscarriages [5]. Porter *et al *concluded that "a specific assay to diagnose immune-mediated early pregnancy loss and a reliable method to determine which women might benefit from manipulation of the maternal immune system are urgently needed" [5]. This trial is intended to make progress towards meeting the challenge of diagnosing and treating immune-mediated early pregnancy loss.

### Uterine Natural Killer Cells

The putative association of recurrent miscarriage and immunological phenomenon has been present for many years. Natural killer (NK) cells which form part of the innate immune system are found in peripheral blood and in the endometrium. Although sharing some similar properties, peripheral and uterine natural killer (uNK) cells are unique cell types with distinct antigenic features and functional markers. UNK cells in peripheral circulation differ in many respects to those in the endometrium [6-8]. It is the intensity of CD56 antigen expression and the lack of two typical NK cell markers - CD16 and CD 57 antigens, that differentiate uterine from peripheral NK cells. Around 80% of uNK cells are CD56^bright ^and CD16^- ^whereas 90% of peripheral NK cells show opposite characteristics; they are CD56^dim ^and CD16^+ ^[7,9].

Uterine NK cells are the most predominant leucocytes in the endometrium and their density varies throughout the menstrual cycle. UNK cell density increases in number towards the mid-luteal phase and peaks in early pregnancy if implantation occurs [8]. UNK cells accumulate as a dense infiltrate at the implantation site near stromal cells, glands, blood vessels and trophoblast cells in early pregnancy [7]. In-vitro studies have also shown that extravillous trophoblast and uNK cells interaction occurs [10]. These interactions may have an effect on trophoblast invasion. However, more recent evidence points to the role of uNK cells as one of controlling angiogenesis [11].

Embryo implantation and early pregnancy development occur in a relatively hypoxic environment (2-3% O_2_) [12]. Inappropriate blood flow to the intervillous space has been associated with oxidative stress damage to the developing placenta and thus miscarriage [13]. UNK cell density in women with recurrent miscarriage was found to be positively correlated with endometrial angiogenesis and uterine artery blood flow [14]. A similar positive correlation was also found in women with unexplained recurrent failure of in-vitro fertilisation (IVF) [15]. Thus, we have proposed that increased uNK cell density is associated with increased number of spiral arteries which may lead to inappropriate blood flow to the developing foetal-placental unit causing oxidative stress and consequent miscarriage [14].

### Uterine NK cells and Recurrent Miscarriage

Increased density of uNK cells in pre-implantation endometrium has been found in women with RM compared to fertile controls [16-19]. However, there are also studies that have shown no difference in the population of uNK cells in patients with RM and controls but these included women with only 2 consecutive miscarriages [20,21]. Whether high numbers of uNK cells in the mid-luteal phase predict subsequent miscarriage is controversial. One study suggested that they do [17] but a more recent slightly larger study refuted this [19]. However, both studies were small in number and were retrospectively analysed.

Studies on normal and miscarried early pregnancy deciduas have also implicated uNK cells in the aetiology of idiopathic RM [22,23].

### The effect of prednisolone on uNK cells

UNK cells express both glucocorticoid and ER-β receptors [24]. Thus, therapeutic manipulation of these cells may be possible. Prednisolone was chosen as the steroid for manipulation as it is metabolised by the placenta and very little of the drug reaches the fetus [25]. One woman with significantly raised uNK cells had 17 miscarriages and was given 5 mg of prednisolone pre-conceptually. She had a further 2 miscarriages and then had 20 mgs of prednisolone once a day both pre-conceptually and in early pregnancy. On this regime, she had a live birth [26].

Next, a prospective study was carried out with 20 mg prednisolone from day 1 to day 21 of the menstrual cycle which demonstrated a reduction in uNK cells in the pre-implantation endometrium of patients with idiopathic RM [18]. This study, carried out in Liverpool investigated 85 women with RM and 18 women with 2 or more normal pregnancies for uNK cells density via mid-luteal phase endometrial biopsy. The normal range of uNK cells was defined using the upper end of the inter-quartile range for the 18 control women. Thus, women with more than 5% uNK cells per stromal cell were considered to have high levels. 32 women with RM had high levels of uNK cells and 29 agreed to take 20 mg prednisolone for 3 weeks and have a second biopsy. In 23 women, the number of uNK cells decreased. The reduction in uNK cell density was significant with a mean level of 14% before treatment to 9% after treatment (p = 0.0004, CI 2.3-12). Furthermore, 3 women requested a third biopsy after a further month of prednisolone and in each case, the uNK cells level had fallen further suggesting that prolonging the steroid therapy for longer than 3 weeks further reduces the level of uNK cells. None of these women reported side effects significant enough to stop the medication.

## Aims of trial

The primary aim of this trial is to investigate whether prednisolone therapy during the first trimester of pregnancy is able to reduce the risk of miscarriage and improve live birth rates in patients with idiopathic RM and raised uNK cells in the endometrium.

### Primary Outcome

The primary outcome is the number of babies born alive.

### Secondary Outcomes

They will include:

- Conception rate

- First and second trimester miscarriages

- Number of losses of empty gestation sacs and foetal losses

- Karyotype of miscarried pregnancies

- Stillbirths

- Intrauterine growth restriction (IUGR) defined as birth weight below the 5^th ^centile according to customised birth weight charts

- Pregnancy complications such as pre-eclampsia or gestational diabetes

- Gestational age at delivery

- Foetal abnormality

- Side effect of steroids (eg: mood changes, weight gain, increased appetite, indigestion, avascular necrosis of the hip, hypertension or hyperglycemia)

## Methodology

### Design

This will be a randomised, double-blind, placebo controlled trial with an initial pilot phase to assess feasibility of recruitment and randomisation. Women are recruited from Liverpool Women's Hospital's recurrent miscarriage clinic which is the tertiary referral centre for the region and an endometrial research clinic. Referrals for the research clinic are from all over UK and Europe following media publicity surrounding the publication of the case report about a successful pregnancy with steroid use after 19 consecutive miscarriages [26] and national presentations.

**Figure 1:**
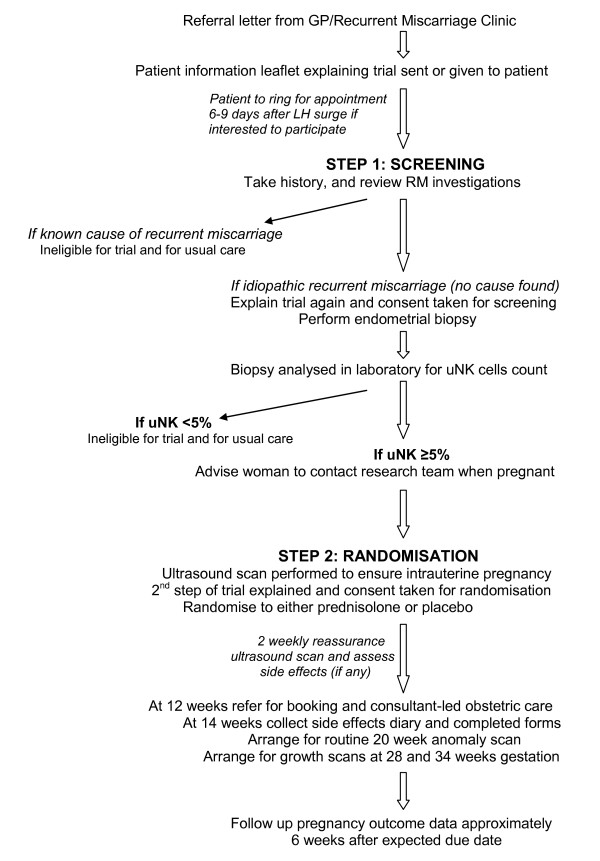
**Patient flow during trial**.

When these women are first seen in the RM clinic, or if a referral letter to the research clinic is received, patient information leaflets regarding the trial are given or sent to them for consideration of their participation in this study (Figure 1). Women willing to participate are encouraged to ring for an appointment 6-9 days after their luteinising hormone (LH) surge in a cycle where they have not tried to conceive (patient information leaflet advises patients to get an ovulation kit and to use contraception). At this appointment, a full history is taken and the results of previous investigations noted to ensure that there is no cause found for RM. The trial is then explained further and consent obtained to perform a transvaginal scan and an endometrial biopsy.

The endometrial biopsy is then taken and the sample fixed in formalin, processed and embedded in paraffin wax. 3 μm sections are prepared and stained for CD56 using immunohistochemistry. A positive control slide from a patient known to have high uNK cells and an IgG mouse negative control is used for every slide undergoing immunohistochemistry to ensure no false negative or false positive staining. Next, 10 high-powered fields from different areas along the epithelial edge of the sample are selected and photographed. Then, the glandular epithelial cells and blood vessel are removed electronically. Subsequently, the number of CD56 positive and negative cells in each field are counted using image analysis software. Image analysis has markedly reduced the inter-observer error as compared with manual counting [27]. For each observer, Bland-Altman plots were produced to ensure that there were no systematic differences in uNK or stromal cell counts between observers. The inter- and intra-observer co-efficients of variation were 12.2 (SD 6.53) and 6.8 (SD 4.29) respectively. The normal range was defined as <5% uNK cells per stromal cell using the upper end of the interquartile range of control patients from a previous study [18]. The positive and negative predictive values will be calculated with the results of the trial. Results of the uNK cell counts are communicated to the women by letter with a follow up telephone consultation. Those with normal uNK cell density are advised to follow standard management. Those with uNK cell counts of ≥5% are advised to contact the research team as soon as they are pregnant to be considered for randomisation.

### Inclusion Criteria

- 3 or more consecutive miscarriages with no cause found (idiopathic)

- Less than 40 years old

- ≥5% uNK cells at day LH +6 to +9

### Exclusion Criteria

- Known cause for recurrent miscarriage: antiphospholipid syndrome (positive anticardiolipin antibody or lupus anticoagulant on 2 separate occasion at least 6 weeks apart), thrombophilia (factor V Leiden mutation, APCR resistance, protein C or S deficiency, prothrombin G20210A mutation, antithrombin III deficiency), abnormal thyroid function tests, parental balanced translocation or uterine anomaly (known subseptate uterus or cervical weakness diagnosed at hysteroscopy).

- Contraindications to steroid therapy: hypertension, diabetes, mental health problems or obesity with BMI >35

- Decline consent to randomisation

### Randomisation and blinding

Women meeting the inclusion criteria are scanned after a positive pregnancy test. If a viable intrauterine pregnancy between 4-8 weeks gestation is found, then a second consent is obtained for randomisation to either prednisolone or placebo. Once consent if obtained, women are given consecutive study numbers then sent to pharmacy. A dedicated pharmacist then allocates to the treatment or placebo group the women using her study number. This study number allocation is performed by using a randomisation list that was pre-constructed with a computerised random number generator in blocks of 20. All women are given a pack of tablets by the pharmacist and advised to take 4 tablets for 6 weeks, 2 tablets for 1 week and 1 tablet for 1 week. Both prednisolone and placebo tablets are similar in size and colour and dispensed in identical packaging. The active tablets consist of 5 mgs of prednisolone and the placebo is an inert substance specially made to be identical to the placebo. Women are also given a chart that tells them how many tablets to take each day and instructions to mark off each day they take the study medication. They are then asked to return this chart to the investigators at 14 weeks gestation so that compliance is monitored. Thus, both the trial investigators and women are blind to the treatment allocation.

Case report forms (CRF) are then completed and given to an independent research administrator who enters the information on a database and keeps them as confidential trial documentation. The research administrator will generate reports to the data monitoring committee (DMC) as necessary.

All women participating in the trial are given a trial ID card where contact details of the hospital are available to get in touch with either the chief investigator (CI) or principal investigator (PI) in the event of an emergency where the trial group allocation needs to be identified. The CI or PI will then authorise the pharmacy department to unblind the group only if severe illness occurs and the attending physician needs to know whether the women had been allocated steroids. The DMC will also be able to unblind these groups if necessary.

### Monitoring in pregnancy

After randomisation, women will be offered a 2 weekly ultrasound scan for reassurance and a clinic consultation to assess side effects of treatment. This will be done by asking them about side effects, reviewing the side effect diary, and measuring the blood pressure until 12 weeks gestation. If hypertension or any significant side effects occurred, they will be asked to stop the medication. Any side effects reported will be assessed in terms of their seriousness, causality and expectedness. If the event was deemed serious, a serious adverse event or adverse event (SAE/AE) report form will be completed by the CI or PI and submitted to the relevant committees. If it was a suspected unexpected serious adverse reaction (SUSAR), then reporting will be expedited accordingly.

At 12 weeks gestation, women are referred to the hospital of their choice for booking and consultant-led antenatal care. At 14 weeks gestation, they are reviewed again and all the CRF are collected. A routine anomaly scan at 20 weeks gestation and growth scans at 28 and 34 weeks gestation will then be arranged. Another telephone follow-up consultation will be arranged 6 weeks after the delivery of the baby to assess the pregnancy and secondary outcomes.

### Safety considerations

Prednisolone is commonly used to treat medical conditions in pregnancy and has been given in the first trimester to women with asthma [28], rheumatoid arthritis [29] and hyperemesis gravidarum [30] with minimal side effects. It is metabolised by the placenta to inactive prednisone and thus only about 10% of active drug reaches the foetus [25]. Although there are concerns of possible complications of intrauterine growth restriction and an association with cleft palate from animal studies, more recent studies, even with postnatal follow-up, have not shown such complications with prednisolone use [30,31].

### Sample size calculation

**Figure 2:**
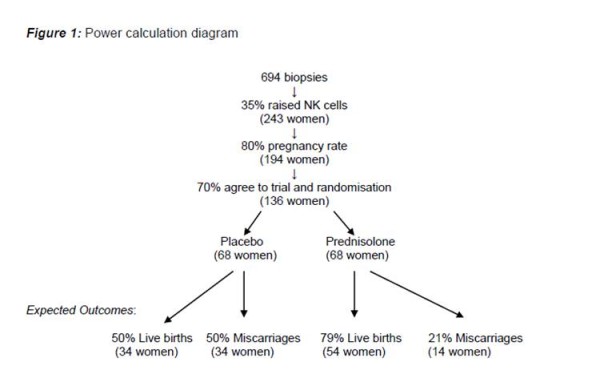
**Power calculation diagram**.

Preliminary power calculation was based on multiple presumptions informed by minimal data. The live birth rate on placebo, is assumed from a previous study where 12 women with >5% uNK cells conceived and 6 subsequently miscarried (50%) [17]. We believe an increase in pregnancy success from 50% to 79% would be clinically important. At present, there is an 80% pregnancy rate in the RM clinic in LWH. Thus, to reach statistical significance, 68 women will need to be recruited in each group (80% power, 5% 2-sided alpha). Assuming a 70% acceptance rate, we will need to ask 136 women to be randomised. Assuming an 80% conception rate after the biopsy and a 35% rate of high uNK cells, 694 patients with idiopathic RM will need to be recruited for endometrial biopsies (Figure 2). The current on-going pilot study can confirm the presumptions made above and provide more information to perform a definitive power calculation in the future.

### Data Analysis

Data analysis will be performed on an intention to treat basis using a data analysis plan that will be finalised before data analysis starts. The outline of the data analysis plan is as follows.

Data will be cleaned prior to any analysis.

The results of the trial, including patient flow during the trial, will be reported in accordance with the guidelines from the CONSORT (Consolidated Standards of Reporting Trials) statement [32].

Analysis will proceed in the following steps:

1. Summary statistics for demographic information relating to the allocation groups will be tabulated. The data will be examined to determine the extent to which the treatment and placebo groups are similar.

2. The primary outcome will be assessed. The live birth rate in each group will be expressed as a risk ratio with 95% Confidence Intervals. Statistical significance will be calculated using the Fisher's exact test. A significance value of p < 0.05 will be considered significant.

3. Secondary outcomes will be assessed. The rates of miscarriage, type of miscarriage (sac loss or fetal loss), karyotype of miscarried pregnancies, conception rate, gestational age at delivery and pregnancy complications will be tabulated. For dichotomous categorical variables, risk ratios and 95% Confidence Intervals will be calculated. For other categorical variables, a chi-squared test will be performed. For continuous variables, non-parametric tests will be used

4. Pre-specified subgroup analyses will include comparisons of women with primary and secondary recurrent miscarriage.

5. Adverse events. All adverse events will be tabulated according to allocation group.

6. Trial process measures. The following will be tabulated: how women come to know of the trial, the rate of acceptance for screening and randomisation, proportion of women who screened high for uNK cells and compliance to medication will be evaluated.

## Trial Management and Monitoring Structure

### Ethical and Regulatory Issues

Approval to conduct the trial has been granted from the Liverpool Local Research Ethics Committee.

The trial is included on the National Institute for Health Research (NIHR) Clinical Research Portfolio (NIHR CRN ID: 6567) and is registered with European and international clinical trials database (EUDRACT No: 2005-003307-36, ISRCTN28090716). MHRA approval was also granted.

### Patient Acceptability and Consent

The trial has been discussed with the President of the Miscarriage Association, as the patient representative. We were advised that women who have had recurrent miscarriages would be suitable to be randomised to a placebo. The Miscarriage Association have also agreed to be independent advisors to women during the recruitment phase and throughout the trial. Additionally, women are informed that if a miscarriage occurred while they are on the trial, they can choose to have prednisolone in the subsequent pregnancy with the understanding that this is yet to be proven as an effective treatment.

Women who fit the inclusion criteria will be informed about the objectives of the study and be given written information about it. They will then have time to consider the trial and contact the investigators for a consultation appointment when they ovulate if interested to participate. Consent will be obtained by the trial investigators prior to screening for levels of uNK cells. A separate consent for randomisation into the study is taken when the woman is found to have raised uNK cells and is pregnant.

### Data Monitoring Committee (DMC)

A data monitoring committee (DMC) independent of the trial investigators has been appointed for the trial to provide independent review of unblinded data at agreed intervals to ensure that no harm is being cause by the treatment. The first DMC meeting will be planned for after 20 patients have been randomised into the trial and passed 14 weeks gestation. Particular emphasis will be placed upon monitoring the side effect of steroids, fertility rate and pregnancy complications.

This committee will also address issues such as:

- Significant problems with trial design or methodology

- Recruitment rate

- Patient's acceptance of the possibility of randomisation to placebo

The chair of the DMC will report to the Trial Steering Committee (TSC) approximately 2 weeks after each meeting in accordance with recommended trial oversight.

### Steering and Management Committees

A trial steering committee (TSC) and trial management group (TMG) have been formed to supervise and manage the trial. The TSC will be responsible for approving the core protocol and any subsequent amendments. The TMG will meet every 4-6 months to discuss:

- the recruitment rate and patient acceptability of the trial

- side effect of steroids

- data collection forms

- potential problems arising from the trial

The TMG will report the above and present any possible solutions to problems or strategies to improve recruitment to the TSC who will meet when deemed necessary by the Chief Investigator.

## Competing interests

The authors declare that they have no competing interests.

## Authors' contributions

SQ conceived the study. AT and SQ participated in the design and coordination of the study and drafted the manuscript. AT, SQ, MT and ZA participated in the management of the study. JD managed the laboratory practice. All authors read and approved the final manuscript.
